# Editorial: Early events during host cell-pathogen interaction, volume II

**DOI:** 10.3389/fcimb.2024.1364415

**Published:** 2024-01-17

**Authors:** Patricia S. T. Veras, Albert Descoteaux, Maria Isabel Colombo, Juliana P. B. de Menezes

**Affiliations:** ^1^ Laboratory of Host - Parasite Interaction and Epidemiology, Gonçalo Moniz Institute, Salvador, Brazil; ^2^ Institut National de la Recherche Scientifique (INRS), Centre Armand-Frappier Sante´ Biotechnologie, Laval, QC, Canada; ^3^ Instituto de Histología y Embriología de Mendoza (IHEM), Consejo Nacional de Investigaciones Científicas y Técnicas (CONICET)-Universidad Nacional de Cuyo, Mendoza, Argentina

**Keywords:** early immune response, trypanosomatids, disease control, disease progression, immunotherapeutic interventions

## Introduction

The parasitic trypanosomatids cause lethal and debilitating diseases such as leishmaniasis, and Chagas disease, with major impacts on human health (Horn, 2022) Continuous research efforts have yielded positive outcomes by elucidating the pathogenesis of these inflammatory diseases and improving treatment. This Research Topic “*Early Events During Host Cell-Pathogen Interaction: Volume II*” includes 3 original research articles in the investigation of early events of host immune response against trypanosomatids infection.

Chagas disease, a chronic illness for which no vaccines or effective treatments are available, also known as American trypanosomiasis, is caused by the parasite *Trypanosoma cruzi* (Pérez-Molina and Molina, 2018). American trypanosomes are primarily transmitted by triatominae bugs, but other significant transmission routes include oral transmission, congenital transmission, as well as through blood transfusions or organ transplantation. It is estimated that over 6 million people worldwide are infected, and up to 30% of them may eventually develop cardiac, digestive, neurological, and potentially life-threatening symptoms, often many years after the initial infection (Horn, 2022). The challenges of treating and monitoring human patients over decades before disease symptoms appear, along with the high costs associated with clinical trials for neglected tropical diseases, have hindered drug development, despite advancements in preclinical research (Morillo et al., 2015; Nunes et al., 2021; Padilla et al., 2022). Similarly, the translation of research findings in drug development into tangible benefits for humans has been delayed due to scientific controversies regarding the mechanisms underlying Chagas disease pathogenesis (De Alba-Alvarado et al., 2023). In this Research Topic, investigated parasitological and inflammatory patterns at the testicular site in mice infected with the Colombian strain of *T. cruzi* while receiving preventive treatment with a novel Theracurmin formulation, rich in the anti-inflammatory natural product, curcumin. The authors showed that the Colombian strain of *T. cruzi* may induce immunological and punctual structural alterations at the testicular site in infected mice, particularly affecting the tunica propria, which is the muscle layer that surrounds the seminiferous tubules, as well as the Leydig cells. Furthermore, the authors also demonstrated that preventive treatment with Theracurmin has a protective effect by regulating the synthesis of IL-6 and IL-15 in the testicular area.

Leishmaniasis is a neglected tropical disease endemic in 98 countries. It is estimated that between 700,000 and 1 million new cases occur worldwide annually, leading to approximately 30,000 deaths (Alvar et al., 2012). This disease results in a wide spectrum of clinical manifestations, ranging from skin lesions at the site of infection to disseminated lesions in internal organs, such as the spleen and liver. Visceral leishmaniasis (VL), the most serious manifestation of leishmaniasis, is a systemic chronic disease caused mostly by *Leishmania infantum* that can lead to death if left untreated (Ashford, 2000; Desjeux, 2004; Kaye and Scott, 2011; Podinovskaia and Descoteaux, 2015). Every year, it is estimated that 50,000 to 90,000 new cases occur, with only 25 to 45% reported to the World Health Organization (World Health Organization, 2022). *Leishmania* infection in the vertebrate host begins when female sandflies inoculate metacyclic promastigotes into the host’s skin during blood feeding (Veras and Bezerra De Menezes, 2016). Once inside the mammalian host, *Leishmania* parasites can survive and multiply inside acidic parasitophorous vacuoles within macrophages (Afrin et al., 2019). Several cells are then recruited to the site of infection, with neutrophils being the first cells to be recruited. To better understand the role of neutrophils in leishmaniasis, Bomfim et al., evaluated the expression of TREM-1 in these cells from patients with VL at different stages of treatment. The authors showed a lower expression of this molecule in the surface of neutrophil from untreated patients and an increase after treatment. In addition, TREM-1 expression was directly correlated with lymphocyte and erythrocyte count, and indirectly correlated with spleen and liver size and IL-22, suggesting that TREM-1 may contribute to the inflammatory imbalance observed in VL patients.

Lipophosphoglycan (LPG) is a major cell surface glycoconjugate on the surface of *Leishmania* promastigotes, which has been shown to play an important role in the parasite–host cell interaction. LPG from *L. donovani* has been linked to a protective effect of the parasite against neutrophilic microbicidal mechanisms, enhancing its survival (Gabriel et al., 2010). However, the role played by this molecule in other species of *Leishmania* is still poorly understood. To investigate the role of *L. infantum* LPG in the context of neutrophil infection, Quintela-Carvalho et al. used an LPG-deficient Δ*lpg1*mutant. They demonstrated that, like *L. donovani*, *L. infantum* LPG promotes intracellular parasite survival associated to reduced neutrophil activation.

In an attempt to better understand the mechanisms involved in the immunopathogenesis of leishmaniasis, Emerson et al. conducted a study on the effects of macrophage infection with *L. donovani* on extracellular vesicle (EV) biogenesis and composition. Using a visceral leishmaniasis (VL) experimental model, the authors demonstrated that parasite molecules are secreted in EVs and can be distributed in infected tissues. They observed an increase in macrophage count, as well as the presence of LdVash, a parasite-derived molecule incorporated into EVs from *L. donovani*-infected macrophages, in the livers of mice infected with *L. donovani*. Finally, the authors found that the EVs produced by macrophages during *Leishmania* infection led to a gene expression profile consistent with M2 polarization.

In summary, this Research Topic provides insights into the early interactions between trypanosomatids and host cells, offering information about recent advances in understanding how host cells respond to infection by these parasites. The study of the immunopathogenesis of diseases caused by trypanosomatids and the mechanisms involved in the immune response and host cells-parasite interaction may, in the future, contribute to the identification of new molecular targets and the development of new therapeutic strategies for these diseases.

## Author contributions

PV: Writing – original draft, Writing – review & editing. AD: Writing – review & editing. JM: Writing – original draft, Writing – review & editing. MC: Writing – review & editing.
